# Genome diversity and evolution of the duckweed section *Alatae* comprising diploids, polyploids, and interspecific hybrids

**DOI:** 10.1111/tpj.70158

**Published:** 2025-04-23

**Authors:** Anton Stepanenko, Luca Braglia, Jörg Fuchs, Veit Schubert, Phuong T. N. Hoang, Yuri Lee, Guimin Chen, Silvia Gianì, Laura Morello, Ingo Schubert

**Affiliations:** ^1^ Leibniz Institute of Plant Genetics and Crop Plant Research (IPK) Gatersleben Seeland 06466 Germany; ^2^ Department of Molecular Genetics Institute of Cell Biology and Genetic Engineering, NASU Kyiv 03143 Ukraine; ^3^ Institute of Agricultural Biology and Biotechnology National Research Council (IBBA‐CNR) Milan 20133 Italy; ^4^ Dalat University Dalat 670000 Vietnam; ^5^ School of Life Sciences Huaiyin Normal University Huai'an 223300 China

**Keywords:** duckweed genus *Lemna*, genomic *in situ* hybridization, interspecific hybridization, phylogeny, plastid and nuclear marker sequences, ploidy level

## Abstract

The section *Alatae* of genus *Lemna* of the monocotyledonous aquatic duckweed family (Lemnaceae) consists of rather diverse accessions with unknown phylogeny and unclear taxonomic assignment. In contrast to other duckweeds, some *Alatae* accessions, in addition to mainly vegetative propagation, produce readily flowers and viable seeds. We analyzed the genomic diversity and phylogenetic relationship of 52 *Alatae* accessions. For this purpose, we applied multiple molecular and cytogenetic approaches, including plastid and nuclear sequence polymorphisms, chromosome counting, genome size determination, and genomic *in situ* hybridization in combination with geographic distribution. We uncovered ploidy variation, recurrent hybridization, and backcrosses between species and their hybrids. The latter successfully spread over three continents. The results elucidate the evolution of *Alatae* accessions and explain the difficult taxonomic assignment of distinct accessions. Our study might be an example for analogous studies to resolve the hitherto unclear relationships among accessions of the duckweed genera *Wolffiella* and *Wolffia*.

## Significance Statement

By means of genome size, plastid and nuclear sequence polymorphisms, chromosome counts, genomic *in situ* hybridization, and geographic distribution, we clarified the hitherto unknown phylogeny of *Lemna* section *Alatae* (duckweeds) and uncovered polyploids, recurrent hybrids, and backcross progenies of the ancestral species *Le. aequinoctialis* and *Le. perpusilla.* Such a complex approach may also apply to resolve evolutionary relationships of the duckweed genera *Wolffiella* and *Wolffia.*

## INTRODUCTION

The aquatic monocotyledonous duckweeds (Lemnaceae Martinov) comprise five genera with so far 35 species and two hybrid species (Bog et al., 2019; Appenroth et al., 2024). Despite their small size and reduced morphology, duckweeds are remarkably diverse and their genetic, physiological and biochemical features, their development, evolution, and practical applications are of increasing interest (for review see Acosta et al., 2021; Cao et al., 2018; Fourounjian et al., 2020). While the phylogeny of the genus *Spirodela* Schleid. with two species, and the monospecific genus *Landoltia* Les & Crawford is obvious, this is not the case for accessions of the remaining genera *Lemna* L., *Wolffiella* Hegelm., and *Wolffia* Horkel ex Schleid. Despite the mainly vegetative propagation of duckweeds, within the genus *Lemna* ploidy variants and so far two hybrid species have been proven: *Lemna × japonica* Landolt (*Le. minor* L. × *Le. turionifera* Landolt, see Braglia, Breviario, et al., 2021; Braglia, Lauria, et al., 2021; Ernst et al., 2025) and *Le. × mediterranea* Braglia & Morello (*Le. minor* × *Le. gibba* L. and reciprocal, see Braglia et al., 2024, Romano et al., 2025).

Among the genus *Lemna*, *Le. aequinoctialis* Welw. (lesser duckweed) has great prospects for practical use as feed for fish, poultry, ruminants, and pigs, as food for humans, as a bioreactor for proteinaceous compounds (Ma et al., 2018; Nati et al., 2024) as well as for bioethanol production (Faizal et al., 2021) and for wastewater remediation (Cai et al., 2022; Hu et al., 2019; Shi et al., 2020; Toyama et al., 2024; Zhou et al., 2018). This is due to its wide geographical distribution in tropical and subtropical climates (Landolt, 1980), and nowadays even in the temperate zones of Europe (Fedoniuk et al., 2022; Vélez‐Gavilán, 2022), its rapid biomass generation, valuable composition, and lack of cytotoxic effects (Nati et al., 2024). The potential for practical use of the sister species *Le. perpusilla* Torr. (minute duckweed) is scarcer due to the small number of available accessions in the public collections and to its limited distribution in the Middle and Eastern states of the USA and Canada (Landolt, 1986; Tippery & Les, 2020). Some researchers claimed its occurrence also for India, China, Korea, and Saudi Arabia (Al‐Dakhil et al., 2021; Halder & Venu, 2012; Lee et al., 2022; Xu et al., 2018). However, the approaches used to identify *Le. perpusilla* outside North America are questionable. Thus, there are only a few reports on its use for phytoremediation (Clark et al., 1981; Tang et al., 2013).

*Le. aequinoctialis* and *Le. perpusilla* are the only currently recognized species in the section *Alatae*, with relatively frequent fruiting. They differ morphologically regarding the number of indistinct ribs of seeds and the seed behavior after ripening (*Le. perpusilla* seeds stay within the fruit wall after ripening, while *Le. aequinoctialis* seeds fall out) (Kandeler & Hügel, 1974; Landolt, 1986). In earlier studies, *Le. aequinoctialis* and *Le. perpusilla* were considered synonymous (Daubs, 1965; Den Hartog & Van der Plas, 1970), but Landolt (1980) recognized them as separate species, as has been confirmed by allozyme studies (Crawford et al., 2001), flavonoid and anatomical‐morphological data (Les et al., 1997), and AFLP (Bog et al., 2010). In addition, some *Alatae* accessions were referred to either *Le. aequinoctialis* or *Le. perpusilla* in different publications, and occasionally, the old name *Le. paucicostata* Hegelm., then synonymized to *Le. aequinoctialis* (Landolt, 1986; Sree et al., 2016), is used for *Alatae* accessions, which caused confusion regarding the identification of these species.

In addition, some researchers distinguish a third species of the *Alatae* section, *Le. aoukikusa* Beppu & Murata, which has been found in Japan. Unlike *Le. aequinoctialis*, *Le. aoukikusa* can survive in colder climates and displays some morphological differences regarding frond thickness, root cap, anther size, flower development, and self‐compatibility (Beppu et al., 1985; Beppu & Takimoto, 1981; Lee et al., 2024). However, it is currently not clear whether the corresponding accessions represent a separate species, a subspecies, or simply a geographically isolated *Le. aequinoctialis* population with variable morphological characteristics.

Therefore, deeper studies of the biodiversity and phylogeny of the *Alatae* section are required. Most of the previous phylogenetic studies of Lemnaceae included only a small number of presumed *Le. aequinoctialis* and *Le. perpusilla* accessions (Bog et al., 2010; Braglia, Lauria, et al., 2021; Tippery et al., 2015), or only specimens collected from geographically restricted areas (Barbosa Neto et al., 2019; Borisjuk et al., 2015; Chen et al., 2022; Lee et al., 2024; Tang et al., 2014, 2015; Wang et al., 2010; Xu et al., 2018). The obtained data were not sufficient for a more comprehensive assessment of the diversity of these species, the more so as *Le. aequinoctialis* accessions are morphologically rather variable (Barbosa Neto et al., 2019; Lee et al., 2024).

Selecting suitable methods to study the diversity and phylogenetic relationship of duckweeds is challenging. Chloroplast barcoding is a relatively inexpensive and easy‐to‐implement method (Borisjuk et al., 2015; Wang et al., 2010). Therefore, it is used in most phylogenetic studies on duckweeds (Barbosa Neto et al., 2019; Braglia et al., 2024; Braglia, Breviario, et al., 2021; Chen et al., 2022; Lee et al., 2024; Tang et al., 2014, 2015; Xu et al., 2018). One of its disadvantages is the inability to identify interspecific hybrids. Another one is the low level of polymorphism between closely related accessions. The latter problem can be overcome by applying multiple marker sequences (Chen et al., 2022). Using nuclear markers is another approach to study the biodiversity of duckweeds. For this purpose, 35S rDNA ITS1 and ITS2 (Tippery et al., 2015), 5S rDNA NTS (Chen et al., 2024), AFLP (Bog et al., 2010), SSR (Xu et al., 2018), genotyping by sequencing (Bog, Xu, et al., 2020), and β‐tubulin gene intron polymorphism (TBP) are used. In particular, TBP provides an easy‐to‐handle, reproducible, and cost‐effective nuclear marker, often informative for resolving intra‐ and interspecific relationships (Braglia et al., 2023; Braglia, Breviario, et al., 2021; Braglia, Lauria, et al., 2021).

Cytogenetic parameters, such as chromosome number and genome size, have proven useful for duckweed taxonomy, highlighting the presence of different cytotypes within the same species (Bog, Sree, et al., 2020; Hoang et al., 2019; Landolt, 1980). In addition, genomic *in situ* hybridization (GISH) confirmed interspecific hybrids (Ernst et al., 2025). All of these approaches have advantages and disadvantages, and no single one alone is of sufficiently informative value (Bog et al., 2022).

Under these circumstances, this study aimed to resolve the phylogenetic relationship of presumed *Le. aequinoctialis*, *Le. perpusilla*, and *Le. aoukikusa* accessions by several independent approaches, including plastid and nuclear markers, genome size, chromosome counts, and GISH, applied to about 50 accessions from different continents and climatic zones.

## RESULTS

### Genotyping by chloroplast barcoding

**Figure 1:**
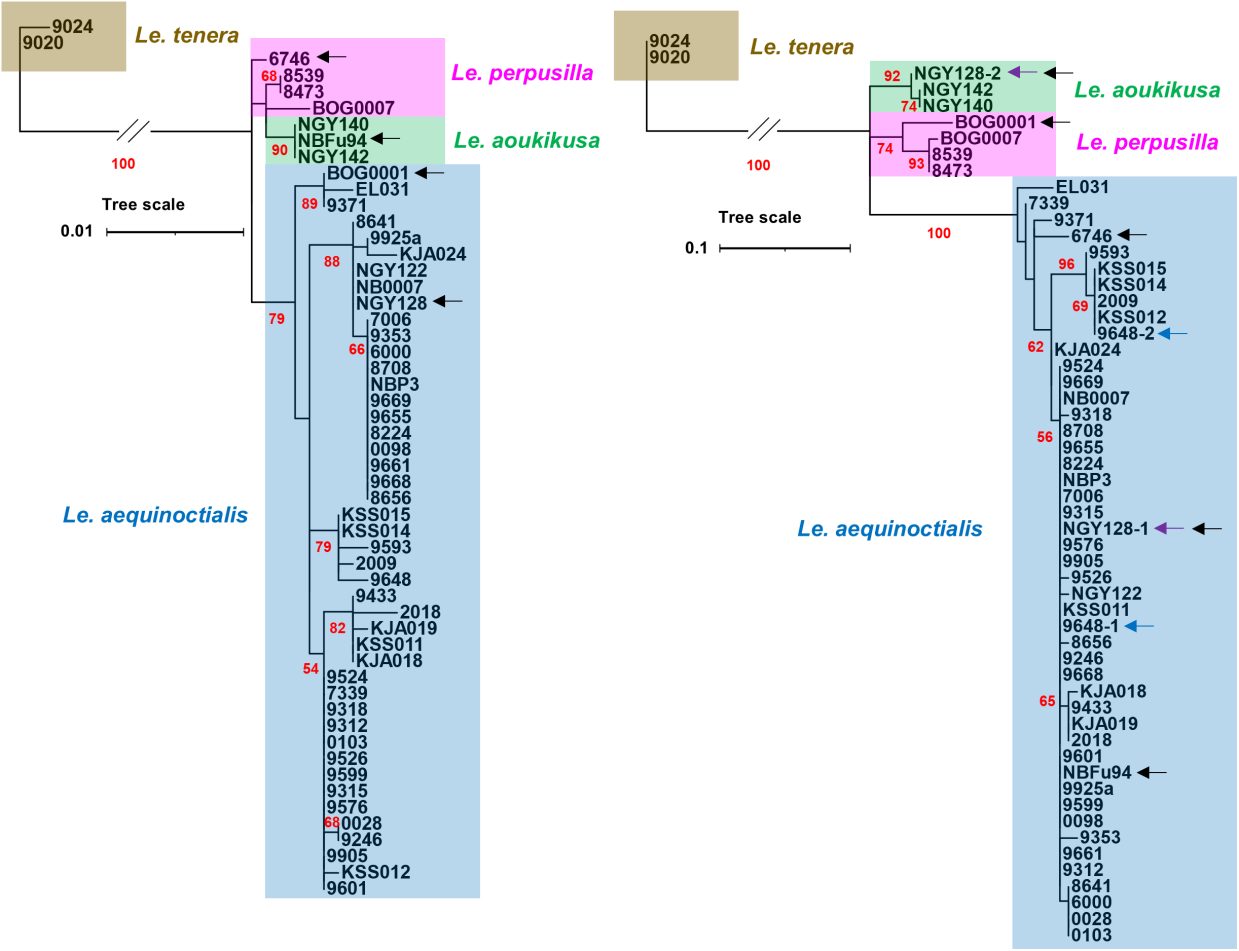
Phylogeny of presumed *Lemna aequinoctialis*, *Le. perpusilla*, *Le. aoukikusa*, and *Le. tenera* accessions based on *atpF–atpH* and *psbK–psbI* intergenic spacer sequences (left) and on ITS1‐5.8rDNA‐ITS2 sequences (right). Black arrows indicate accessions with different positions in barcoding and ITS trees; colored arrows indicate accessions for which different ITS sequences were found. Bootstrap values >50 are shown by red numbers at the branches.

We genotyped 52 *Alatae* and two *Le. tenera* Kurz accessions by direct sequencing of the chloroplast gene spacer sequences of *atpF‐atpH* and *psbK‐psbI* (Figures S1 and S2). Since the analyses of single spacers did not give unambiguous results, we combined *ATP* and *PSB* analyses using *Le. tenera* as an outgroup species. To confirm the identity of *Le. tenera*, we sequenced the *atpF‐atpH* spacers for accessions 9020 and 9024. The *psbK–psbI* spacers were obtained from NCBI (accession numbers KJ136049 and KJ136048, respectively) for the same *Le. tenera* accessions. The combined data matrix for *Le. aequinoctialis*, *Le. perpusilla*, and *Le. aoukikusa* contained 972 characters, divided into two partitions: 1–465 for *atpF‐atpH* and 466–972 for *psbK‐psbI*, of which 892 were constant, 17 variable characters were parsimony uninformative, and 63 were parsimony informative. The combined data matrix for all tested accessions, including *Le. tenera* as an outgroup contained 847 constant, 21 variable but parsimony uninformative, and 104 parsimony informative characters. The resulting phylogenetic tree is shown in the left part of Figure 1.

*Le. aequinoctialis* clones form a distinct cluster with several subclusters. Notably, one of the subclusters contains BOG0001, which previously was assigned to *Le. perpusilla* based on tubulin gene polymorphism (Braglia, Lauria, et al., 2021). In addition to this accession from the South‐Eastern USA, the subcluster contains 9371 and EL031 from Venezuela and Brazil, respectively (for geographic origin of accessions see also Figure 6; Table S1).

The remaining accessions form clusters with acceptable bootstrap support. One contains two presumed *Le. aoukikusa* accessions from East Asia (NGY140, NGY142) and NBFu94, originally classified as *Le. aequinoctialis*; another one three *Le. perpusilla* accessions from the Eastern USA of which BOG0007 differs from the other two, while the presumed *Le. aequinoctialis* accession 6746 from the western part of the USA forms a separate branch. This clone shares five specific SNPs/indels with *Le. perpusilla* and only two SNPs with *Le. aequinoctialis*.

### Genotyping by ITS1 and ITS2

To amplify the variable spacer sequences between the 18S and 5.8S rDNA (ITS1) and between 5.8S rDNA and 25S rDNA (ITS2), we designed a set of primers based on published 18S and 25S rDNA of Lemnaceae. The cloned and sequenced ITS1‐5.8S‐ITS2 regions for 52 *Alatae* and two *Le. tenera* accessions as outgroup (Figure S3) were evaluated.

The combined data matrix for *Le. aequinoctialis*, *Le. perpusilla*, and *Le. aoukikusa* included 724 characters divided into three partitions: 1–307 for ITS1, 308–465 for the 5.8 rRNA gene, and 466–724 for ITS2, of which 662 were constant, 19 were variable but parsimony uninformative, and 43 were parsimony informative. The combined data matrix for all accessions, including *Le. tenera* as an outgroup, contains 589 constant characters, 15 variable but parsimony uninformative, and 120 parsimony informative characters. The resulting phylogenetic tree is shown in the right part of Figure 1.

Bioinformatic and phylogenetic analyses revealed compelling differences between the ITSs of *Le. aequinoctialis*, *Le. perpusilla*, *Le. aoukikusa*, and *Le. tenera* accessions. The tested accessions displayed only one type of ITS1 and ITS2, except for 9648 and NGY128, which showed two types of ITS sequences, suggesting their hybrid nature (9648 intraspecific between different *Le. aequinoctialis* accessions, and NGY128 between *Le. aequinoctialis* and *Le. aoukikusa*).

Apart from the outgroup accessions of *Le. tenera*, the two *Le. aoukikusa* accessions and NGY128 formed a separate cluster, distinct from those of *Le. aequinoctialis* and *Le. perpusilla*, but closer to *Le. perpusilla*. It should be noted that NBFu94 from China, assigned to *Le. aoukikusa* according to chloroplast barcoding results, belongs to *Le. aequinoctialis*, but its ITS marks it as another hybrid candidate, a potential backcross of *Le. aoukikusa* with *Le. aequinoctialis* as paternal ancestor. Because this clone is no longer available, no further investigation was possible. Remarkably, also the presumed *Le. aequinoctialis* accession 6746 and the presumed *Le. perpusilla* accession BOG0001 showed discordant results by chloroplast barcoding and ITS genotyping. Thus, they are also hybrid candidates. That sometimes ITS sequences are not detected from both presumed parental ancestors could be due to frequent uniparental loss of one ancestor's rDNA.

Three *Le. perpusilla* accessions and BOG0001 (assigned to *Le. aequinoctialis* by barcoding) formed a distinct cluster, with BOG0001 branching apart from the other three.

The presumed *Le. aequinoctialis* accessions form a separate, rather homogeneous cluster with strong support. Notably, three American clones, 6746, 9371, and EL031, belong to this cluster but are quite variable and different from the main group of *Le. aequinoctialis*.

To further study discrepancies between barcoding and ITS trees as well as to confirm potential hybrids, we measured genome sizes, counted chromosomes, and performed GISH experiments.

### Genome size measurements

**Figure 2:**
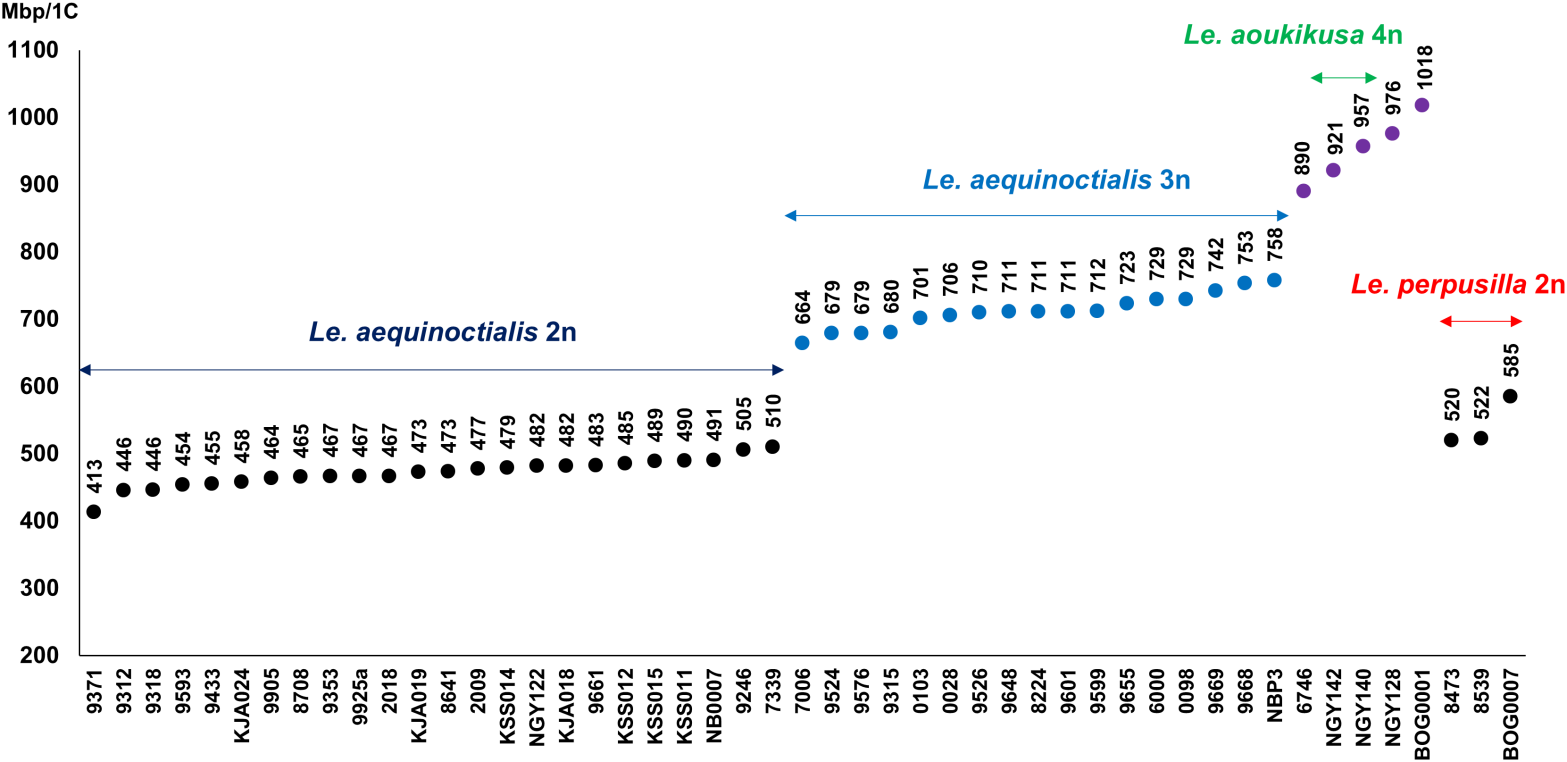
Genome size measuring revealed potentially diploid (black), triploid (blue) and tetraploid (violet) *Alatae* accessions.

According to their flow‐cytometrically determined genome size, the presumed *Le. aequinoctialis* accessions are divided into distinct groups (Figure 2; Table S1). The first largest group contains 24 clones with a genome size ranging from 446 to 510 Mbp, except for accession 9371 with only 413 Mbp. The second group consists of potentially triploids, displaying a genome size variation of 14%. To the accessions of triploid genome sizes belongs 9648, suspected to be an intraspecific hybrid based on two ITS variants. The three diploid *Le. perpusilla* accessions (8473, 8539 and BOG0007) have deviating genome sizes of 520, 522, and 585 Mbp, respectively. The five potentially tetraploid clones vary also regarding their genome size, ranging from 890 Mbp for 6746 to 1018 Mbp for BOG0001. Both are potential hybrids according to discordant results in chloroplast barcoding and ITS genotyping. The remaining three tetraploid accessions (NGY142, NGY140 and NGY128) were previously assigned to *Le. aoukikusa* or to *Le. aequinoctialis* (NGY128), respectively (Lee et al., 2024).

To confirm the ploidy variants, some accessions were selected for chromosome counting. To test for potential allopolyploidy, tri‐ and tetraploid accessions were submitted to GISH.

### Chromosome counting

We selected for chromosome counting diploid accessions of *Le. aequinoctialis* (NB0007, 9925a) and *Le. perpusilla* (BOG0007, 8473), triploid (9648, 9668, 7006, 9526) and tetraploid (BOG0001, NGY142, 6746, NGY128) *Alatae* accessions from different groups, based on molecular marker diversity. The number of chromosomes was counted on at least three metaphases for each selected accession.

**Figure 3:**
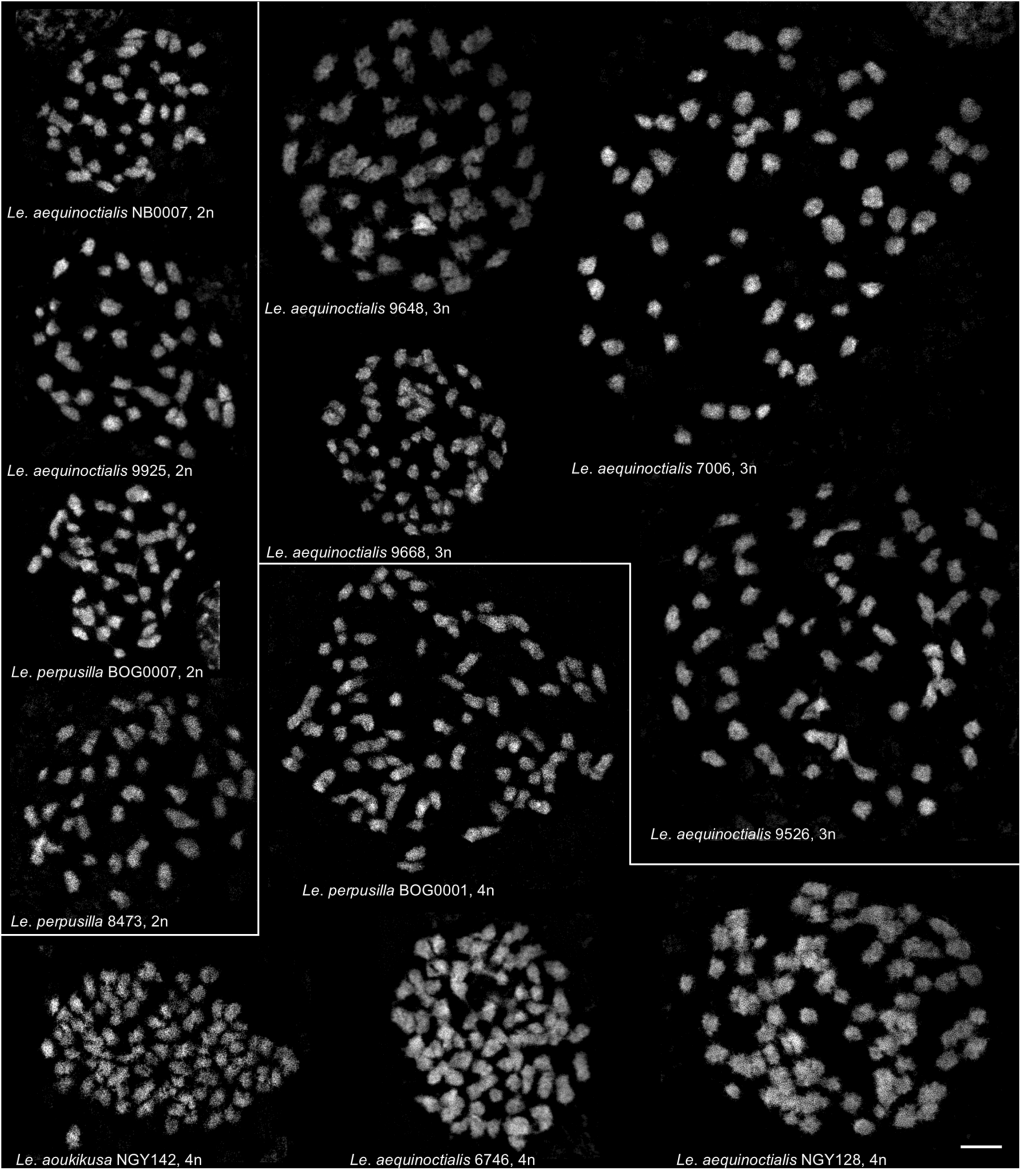
Chromosome spreads of selected diploid (2*n* = 42), triploid (3*n* = 63), and tetraploid (4*n* = 84) *Alatae* accessions. Scale bar = 3 μm.

The diploid accessions displayed invariably 42 predominantly small chromosomes (2*n* = 42) after DAPI staining. These results are consistent with previous counts for the *Le. aequinoctialis* accession 2018 (Hoang et al., 2019). Sixty‐three chromosomes were counted for all tested triploid accessions and 84 for tetraploid ones (Figure 3). These data reflect the previously reported variability of chromosome number for *Le. aequinoctialis* (for review see Hoang et al., 2022). Intermediate numbers, such as 50, 60, 70, and 80 chromosomes as previously reported for some *Le. aequinoctialis* accessions (Urbanska, 1980) are not supported. Because we had previously re‐counted some accessions used by Urbanska and obtained different results (Hoang et al., 2022), the differences (of which 50 and 70 make no sense biologically) were most likely due to miscounting by Urbanska. Due to their small size and often lacking structural details, such as primary (centromere) or secondary (nucleolus‐organizing region [NOR]) constrictions, the chromosomes of the accessions studied here were barely individually distinguishable. It remains an open question whether they are mono‐ or holocentric, albeit ChIP‐sequencing using anti‐CENH3 antibodies revealed monocentricity for chromosomes of the section *Lemna* (Ernst et al., 2025).

### Genomic *in situ* hybridization (GISH)

**Figure 4:**
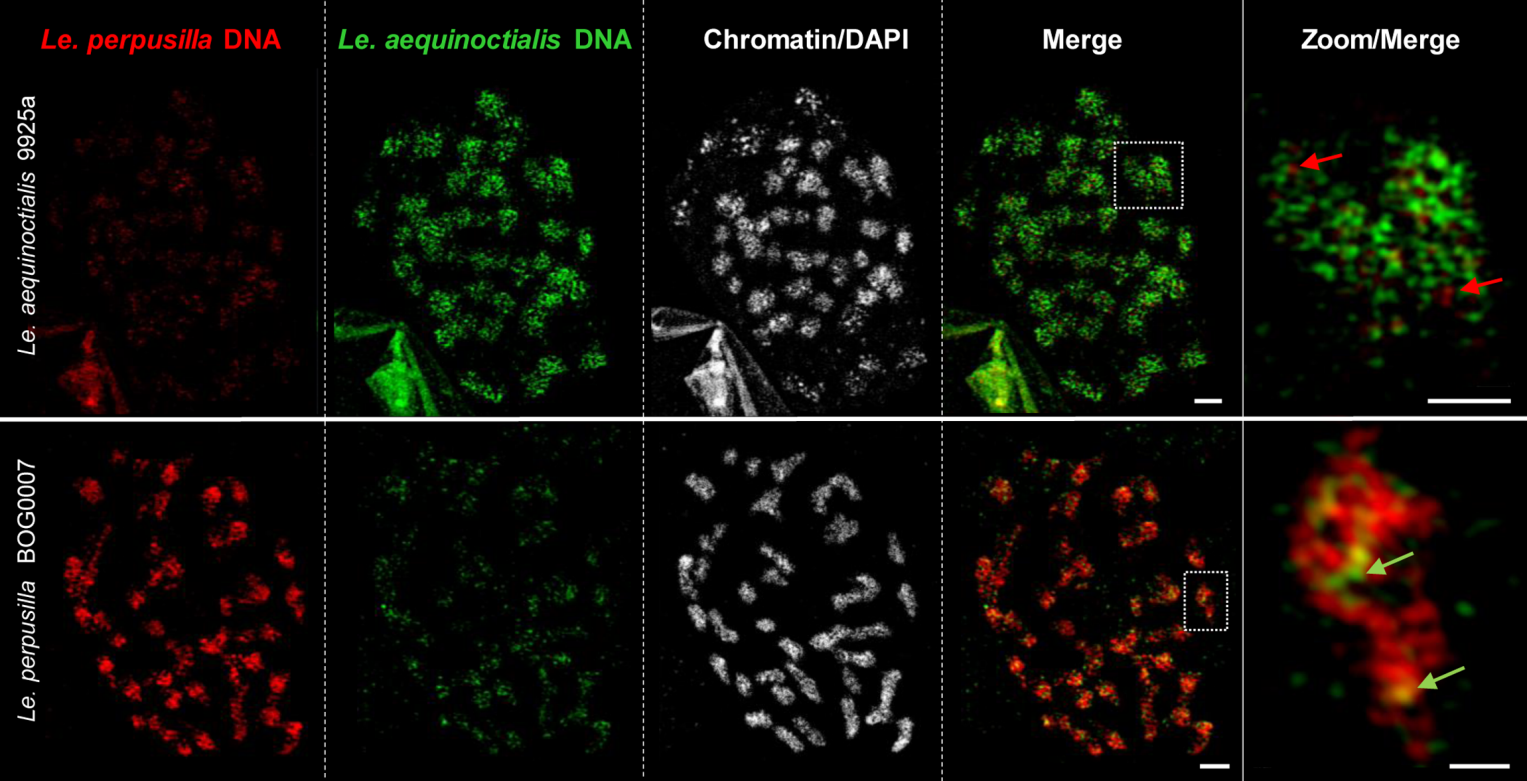
Fluorescence *in situ* hybridization with genomic DNA of *Le. perpusilla* (red) and *Le. aequinoctialis* (green) on mitotic chromosomes of diploid *Le. aequinoctialis* 9925a and *Le. perpusilla* BOG0007. The enlarged insets show cases of cross‐hybridization (arrows). Bars in complete metaphase cells: 2 μm; in enlarged regions: 0.5 μm.

For the analysis of potential allopolyploidy, GISH with genomic DNA of the potential parental taxa has been employed to label chromosomes of suspected hybrids. As a control, GISH was performed with differently labeled genomic DNA from two diploid accessions, *Le. aequinoctialis* 2018 and *Le. perpusilla* BOG0007, against the chromosomes of both accessions (Figure 4). This resulted, as expected, in specific labeling of the chromosomes by the DNA of the corresponding accession. A low level of cross‐hybridization was observed (see zoomed inserts in Figure 4).

**Figure 5:**
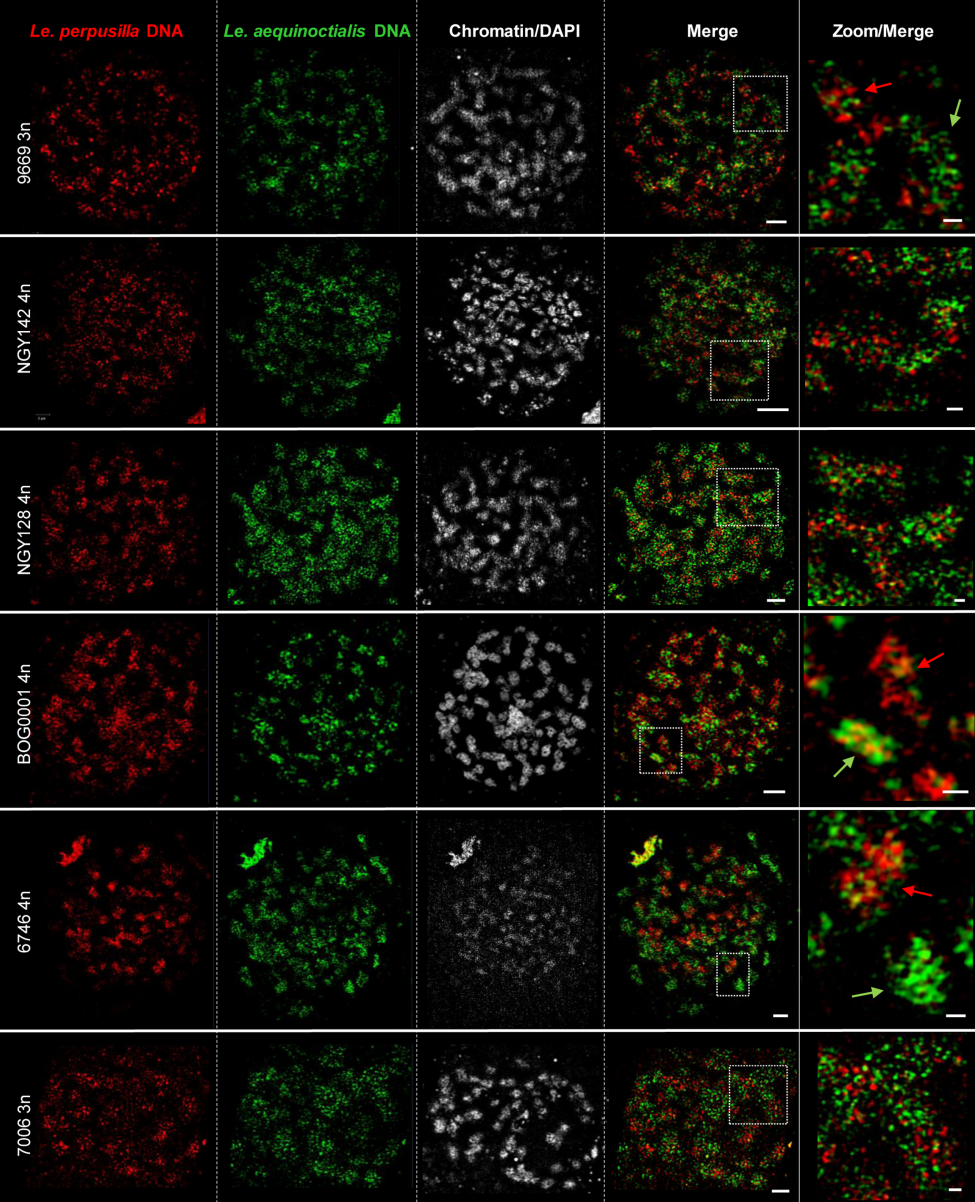
Mitotic metaphases of tri‐ and tetraploid accessions after GISH using genomic *Le. perpusilla* (red) and *Le. aequinoctialis* (green) DNA and applying SIM. The intensity of red and green signals counted in SIM images was close to a 1:1 ratio for exemplarily counted accessions NGY142, NGY128, and 6746. The enlarged regions show cross‐hybridizations (arrows) in predominantly red or green labeled chromosomes. Bars in complete metaphase cells: 2 μm; in enlarged regions: 0.5 μm.

GISH was then performed on triploid (9669, 7006) and tetraploid accessions (NGY142, NGY128, BOG0001, 6746), the latter three suspected to be hybrids based on discordant barcoding and ITS data (Figure 1). All of them were confirmed as interspecific hybrids between *Le. aequinoctialis* and *Le. perpusilla* (Figure 5), including NGY142, which was described previously as fertile *Le. aoukikusa* (Lee et al., 2024).

Cross‐hybridization, that means red signals on predominantly green chromosomes and *vice versa*, suggests somatic recombination between homeologs during double‐strand break (DSB) repair via gene conversion as evidenced between sister chromatids of the field bean (Schubert et al., 2011). The cross‐hybridization frequency likely increases with the age of the hybrid.

### Genotyping by tubulin‐based polymorphisms (TBP)

**Figure 6:**
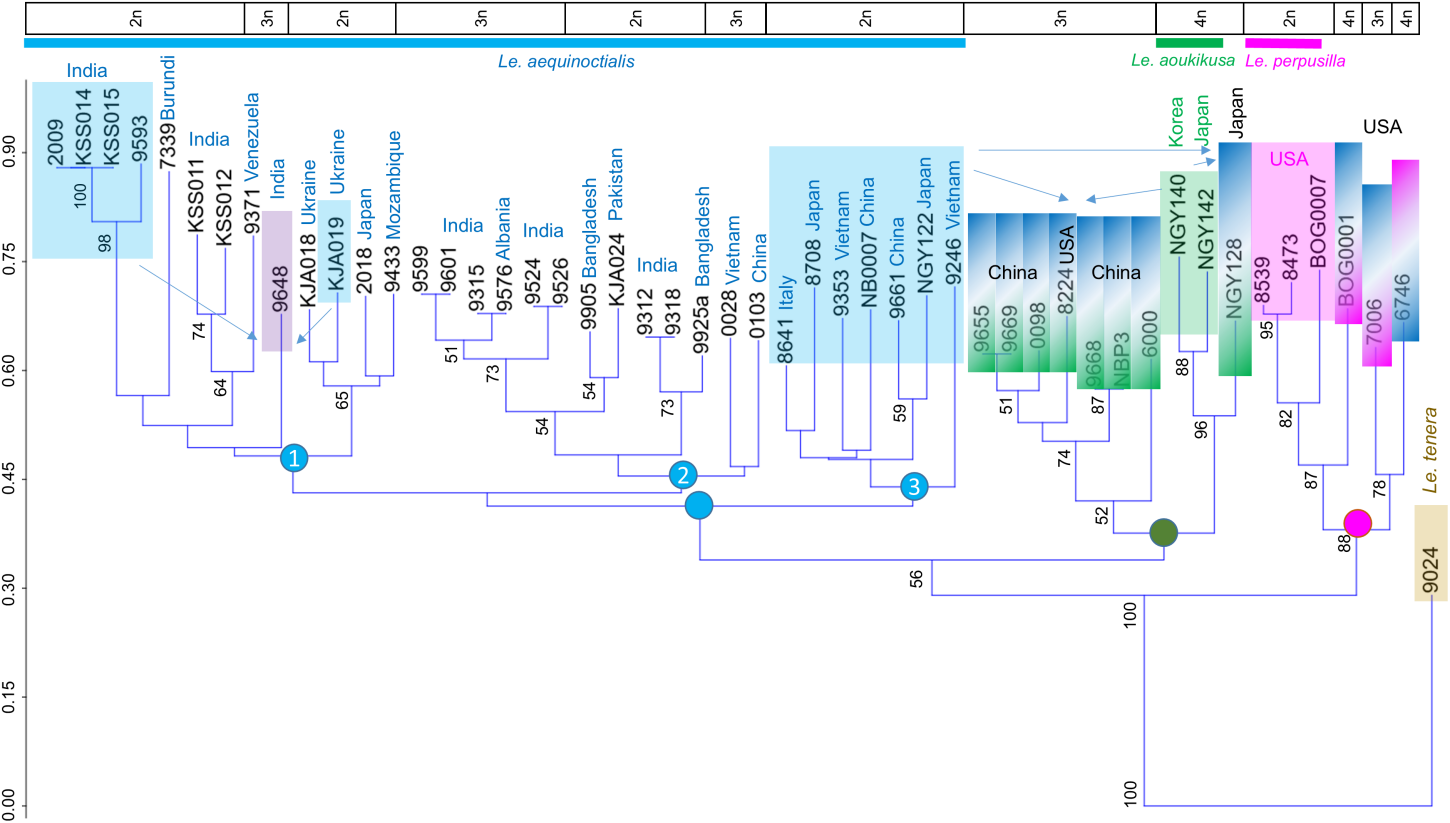
Phylogeny of presumed *Lemna aequinoctialis*, *Le. perpusilla*, *Le. aoukikusa*, and *Le. tenera* (as an outgroup) accessions based on tubulin gene intron polymorphisms. Colored dots mark clades of *Le. perpusilla sensu stricto* and its recent hybrids with *Le. aequinoctialis* (pink dot), the ancient tetraploid hybrids between *Le. aequinoctialis* and *Le. perpusilla*, *Le*. × *aoukikusa*, and their triploid backcross hybrids with diploid *Le. aequinoctialis* (green dot), and *Le. aequinoctialis s.s*. and its autotriploid accessions and intraspecific hybrids (blue dot). In the upper part, ploidy level and geographic origin of the accessions are indicated. Arrows and/or color gradient indicate potential ancestors of hybrids; color gradients mark maternal (top) versus paternal (bottom) ancestors.

The combined data for genome size, chromosome number (both indicating the ploidy level), GISH, and geographic origin are integrated into the phylogenetic tree derived from TBP analyses for the *Alatae* accessions (Figure 6). At its first node from the root, the TBP tree clearly separates the clade including three diploid *Le. perpusilla s.s*. accessions and the relatively young (compared to *Le. aoukikusa*) tri‐ (7006) and tetraploid (BOG0001 and 6746) hybrids with *Le. aequinoctialis* (as confirmed by GISH) (pink dot) from all other clones. Accessions 7006 and BOG0001 have *Le. aequinoctialis*, and 6746 has *Le. perpusilla* as a maternal parent, as suggested by chloroplast barcoding.

A second node separates the *Le. aequinoctialis s.s*. clade (blue dot) from a clade including its ancient tetraploid hybrids with *Le. perpusilla*, *Le. aoukikusa* (NGY140, NGY142), and the tri‐ and tetraploid backcross accessions with *Le. aequinoctialis* as the maternal parent (green dot), according to barcoding results (Figure 1). The allotriploids are supposed to have received the *Le. perpusilla* genome through hybridization of *Le*. *aoukikusa* with diploid *Le. aequinoctialis*, as suggested by their closer proximity to *Le. aoukikusa* than to *Le*. *perpusilla*. For the tetraploid accession NGY128, hybridity between *Le. aequinoctialis* (as plastid donor) and *Le. aoukikusa* is supported by the heterozygous ITS and confirmed by GISH and TBP. Shared introns between hybrids and the parental species are highlighted in Table S3. For the older tetraploid hybrid accessions NGY140 and NGY142 between *Le. aequinoctialis* and *Le. perpusilla*, the plastid sequences are different from both parents but closer to the latter. This suggests a long‐lasting, independent evolution of this lineage. This is also in agreement with some exclusive ß‐tubulin alleles, diverging from both parental species (*Le. aequinoctialis* and *Le. perpusilla*) but shared by all hybrid accessions in this cluster (see Table S3).

Among *Le. aequinoctialis s.s*. (blue dot in Figure 6), we distinguish three major subclusters, without strong support by bootstrap values (<50%), but coincident with barcoding and ITS results. Subcluster 1 includes diploid *Le. aequinoctialis* accessions and a single triploid one (9648). According to TBP and ITS analyses, accession 9648 is heterozygous and merges alleles of diploid accessions from two branches of the same subcluster (Figures 1 and 6; Table S3), as result of an intraspecific cross. Subcluster 2 includes diploid and autotriploid accessions because plastid, ITS, and tubulin intron sequences are all from *Le. aequinoctialis*. Subcluster 3 exclusively comprises diploid accessions.

The allotriploid hybrids vary as to their genome size apparently depending on which *Le. aequinoctialis* and *Le. perpusilla* accession were the actual parents (Figure 2). For instance, 8224 (711 Mbp) could have obtained one genome of a paternal accession like 8473 (520 Mbp) and two maternal *Le. aequinoctialis* genomes of ~450 Mbp. The differing genome sizes of the tetraploid hybrids are also explainable by combinations of correspondingly variable parental genomes. No polyploid hybrid had a larger genome size than expected from the combination of potential parental accessions. This is reasonable, because interspecific hybridization results in loss rather than gain of genetic material (for review see Wang et al., 2021).

## DISCUSSION

### The evolution of the *Alatae* section

Our data demonstrate hybridity and polyploidy as the reasons for the high variability of genome size, chromosome number, morphological features, and for the discordance of phylogeny by chloroplast and nuclear markers among *Alatae* accessions. This variability is responsible for the difficulty of assigning individual accessions to distinct taxa. Because of the relative easiness and frequency of fusion of reduced and/or unreduced gametes from the same or related, but different taxa within the section *Alatae*, recurrent ploidy variants and hybrids appear despite the predominantly vegetative reproduction via sprouting of “daughter” fronds from the meristematic pockets of the “mother” fronds. Auto‐ and allotriploids, resulting from the merge of a reduced with an unreduced gamete, most likely propagate exclusively vegetatively. However, the allotetraploids retained (or re‐gained) the ability to reproduce sexually and to hybridize further as suggested by the allotriploids of the *L. aoukikusa* cluster (Figure 6), which apparently descended from a haploid female gamete of *Le. aequinoctialis* and a diploid male gamete of the allotetraploid hybrid *Le. aoukikusa*.

The allotetraploids 6746 and NGY142 were previously demonstrated to be self‐fertile (Lee et al., 2024) but their hybrid nature was not known. So far, fertile allotetraploid duckweed hybrids are for the first time demonstrated here, but possibly also occur within the *Wolffia* genus. In contrast to the hitherto described hybrid species *Le. × japonica* (Ernst et al., 2025) and *Le. × mediterranea* (Braglia et al., 2024), some *Alatae* hybrids are tetraploid and fertile, while dihaploid hybrids were not yet found among *Alatae* accessions. As described for *Le. × mediterranea* (Braglia et al., 2024), multiple independent hybridizations have occurred also among *Alatae* species. The previously claimed species *Le. aoukikusa* (Lee et al., 2024) represents such a hybrid (see Figures 1 and 6). In addition, the iconic clone 6746, collected in 1954 in California and used for years in several experimental works, was first classified as *Le. perpusilla* by Landolt, and later emended to *Le. paucicostata* and finally ended up with the name *Le. aequinoctialis* (Landolt, 1986). Our demonstration of the hybrid origin of 6746 not only clarifies the reason for the uncertainty of its species assignment, but most likely explains also the reason for its homogamy and self‐fertility, which contrasts with the protogynous and self‐sterile flowers of diploid *Le. aequinoctialis* (Lee et al., 2024). The same reproductive style is found in the *Le. aoukikusa* accession NGY142 (Lee et al., 2024), which is also a hybrid between *Le. aequinoctialis* and *Le. perpusilla*.

**Figure 7:**
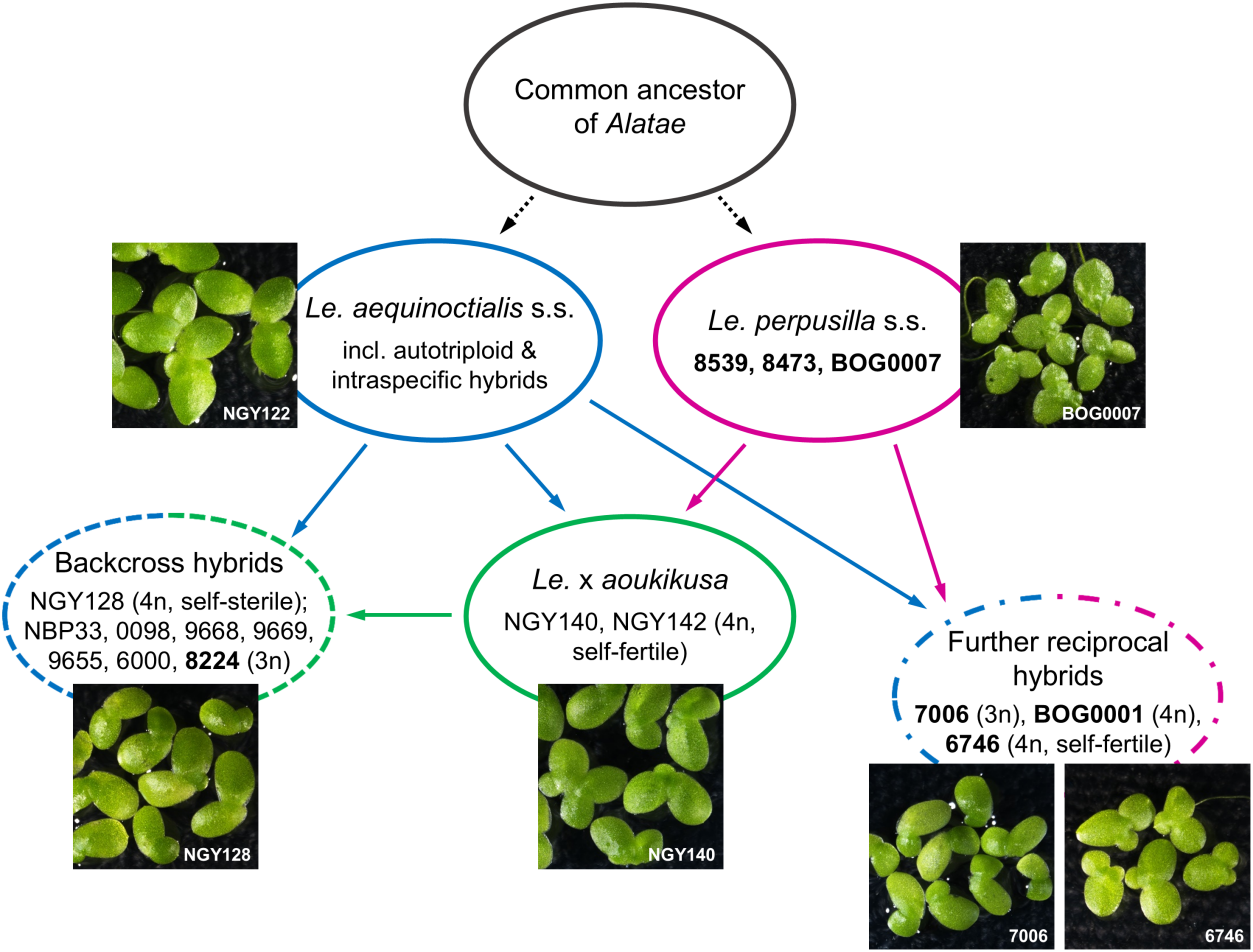
Hypothetical scenario of evolution within *Lemna* section *Alatae*. In bold are accessions from America. The classically defined diploid species *Le. aequinoctialis* and *Le. perpusilla sensu stricto* were confirmed by the applied molecular methods. The previously named *Le. aoukikusa* accessions were uncovered as tetraploid interspecific hybrids between *Le. aequinoctialis* and *Le. perpusilla*. In addition, further tri‐ and tetraploid hybrids as well as tri‐ and tetraploid “Backcross hybrids” were detected in the USA and China, respectively. All duckweed images at fourfold magnification.

Because tri‐ and tetraploid *Alatae* hybrids occur in Northern America and in Asia as well, but there is not yet unambiguous evidence for *Le. perpusilla* outside of Northern America, it seems reasonable to assume the interspecific hybrids occurred in Northern America and some of them successfully spread to Asia. Thus, the resolution of the complex *Alatae* section led to the following hypothetical scenario (see Figure 7):

Ancestors of the related and sexually proficient species *Le. aequinoctialis* and *Le. perpusilla*, with the ability to form unreduced gametes in addition to reduced ones, occurred together in the southern part of North America, where they hybridized repeatedly by fusion of unreduced gametes. The primary hybrid *Le. aoukikusa* migrated towards East Asia due to its adaptation to lower temperatures. There, *Le. aoukikusa* itself backcrossed with *Le. aequinoctialis* (forming the allotriploid branch of the *Le. aoukikusa* cluster in Figure 6). One of the triploid hybrids, 8224, possibly migrated to the United States. In parallel, autotriploids and intraspecific hybrids of *Le. aequinoctialis* arose in various geographic regions by fusion of reduced with unreduced gametes and spread there successfully. The reciprocal hybridization between *Le. aequinoctialis* and *Le. perpusilla*, forming tri‐ and tetraploid hybrids in North America (Figure 6) is apparently ongoing.

### Taxonomy of *Alatae*

An open question remains how to treat the *Alatae* accessions taxonomically. In addition to *Le. aequinoctialis s.s*. and *Le. perpusilla s.s*., we have the allotetraploid *Le. aoukikusa*, as well as a bunch of other tri‐ and tetraploid hybrids involving *Le. aequinoctialis* and *Le. perpusilla* or *Le. aoukikusa* (in the *Le. aoukikusa* and *Le. perpusilla* clusters see Figure 6) for which the taxonomic status remains unclear. Although the systematics of Lemnaceae has been extensively studied (Bog et al., 2019; Landolt, 1986; Les et al., 1997), only a few considered the *Alatae* section, due to its complexity. Among them, recognition of *Le. perpusilla* as a separate species (Les et al., 1997) and the acceptance of *Le. aequinoctialis* as a correct name (Landolt, 1986; Sree et al., 2016). However, even now, this has not led to the termination of the use of the old nomenclature *Le. paucicostata* instead of the generally accepted one, which introduces additional confusion in the taxonomic handling of *Alatae* accessions (16 references in pubmed.ncbi.nlm.nih.gov in the last 10 years). The variability of the morphological characteristics of *Alatae* accessions led to the use of different key criteria for species determination in different studies (Bog, Appenroth, & Sree, 2020; Landolt, 1986; Lee et al., 2024; Sree et al., 2016).

Our molecular and cytogenetic analyses confirmed two diploid species but revealed distinct subgroups with a high level of variability among them. This correlates with previous studies indicating significant variation within the *Alatae* section (Chen et al., 2022; Lee et al., 2024; Tang et al., 2014, 2015). We also specified the genetic structure and geographic distribution of the previously presumed species *Le. aoukikusa* (Beppu & Takimoto, 1981; Lee et al., 2024) as an ancient tetraploid hybrid between *Le. aequinoctialis* and *Le. perpusilla* for which no diploid genotypes are known. Its self‐fertility opens the possibility to consider it as a hybrid species, thus an evolving lineage, rather than a dead end as dihaploid and triploid hybrids usually are. On the other hand, the highlighted complexity clearly indicates we are facing a taxonomically complex group (TCG; Ennos et al., 2005) in which the boundary of species blurs due to the presence of uniparental reproduction, high rate of intercrossing, backcrossing, and polyploidization. This generates a genetically diverse mixture of related individuals of more than one ploidy level, whose biological diversity defies simple classification into discrete species, according to the biological species concept (Ennos et al., 2005). Well‐studied examples are found in the *Senecio* and *Sorbus* genera (for reviews see Robertson et al., 2010; Wong et al., 2022). Assignment of a specimen to a species or a hybrid is possible only by a combination of approaches. Based on the available data we consider the *Alatae* accessions as *Le. aequinoctialis* species complex including allopolyploid hybrids with *Le. perpusilla*.

Summarizing, the data obtained allowed a detailed assessment of the *Alatae* section from different continents using molecular and cytogenetic methods. A high level of diversity, the presence of ploidy variants, and interspecific hybrids were demonstrated. The latter resulted from the fusion of unreduced (or reduced and unreduced) gametes of different species and explains the taxonomic uncertainty within the section. Our results exemplify how the evolution of species of the genera *Wolffiella* and *Wolffia* of the duckweed family could be resolved in the future.

## MATERIALS AND METHODS

### Plant material

We included in our study 52 duckweed accessions from different continents (Europe, Asia, America and Africa). The full list of accessions and research conducted on them is given in Table S1. The analyzed *Le. aequinoctialis*, *Le. perpusilla*, and *Le. aoukikusa* accessions were kindly provided by Klaus Appenroth, Eric Lam, Nicolai Borisjuk, Manuela Bog, and Sowjanya Sree. The accessions were sterilized as described (Appenroth, 2023). The sterile clones were maintained on solid N medium (Appenroth et al., 1996) with 0.6% of Gelrite. To obtain plant material for DNA extraction and metaphase chromosome preparation, duckweeds were grown on a liquid SH medium (Schenk & Hildebrandt, 1972) with 0.5% sucrose. For some accessions which meanwhile were lost from collections (EL031, NBFu94, 8656), DNA preparations were preserved and included in this study for analyses of plastid and ITS polymorphisms.

### DNA extraction and chloroplast barcoding

Total DNA was extracted from plant tissue using a modified CTAB method (Murray & Thompson, 1980). The PCR amplification for barcoding was carried out as recommended by the CBOL Plant Working Group (2009), described in Wang et al. (2010), using specific primers for the *atpF‐atpH* and *psbK–psbI* barcode. Following amplification, the DNA fragments were purified using the NucleoSpin Gel and PCR Clean‐up Kit (Macherey‐Nagel, Düren, Germany) and sequenced using a custom service provided by LGS Genomics GmbH (Berlin, Germany). The obtained forward and reversed sequences were assembled and analyzed using the CLC Main Workbench (Version 6.9.2; Qiagen) software. The obtained sequences were deposited in GeneBank (for accession numbers see Table S1).

### Cloning and sequencing of ribosomal RNA genes

For analysis of the ITS1‐5.8S‐ITS2 region of *Alatae* accessions, the specific DNA fragments were amplified by PCR from the same samples of total DNA as for barcoding. In the standard PCR, we used two primers (18S‐F1 and 25S‐R1) specific for 18S and 25S rDNA (Table S2), respectively. The protocol was optimized to amplify GC‐rich regions (Stepanenko et al., 2022). After cutting out the gel with PCR products and DNA purification using NucleoSpin Gel and PCR Clean‐up Kit (Macherey‐Nagel), the generated fragments were cloned into the vector pMD19 (Takara, Dalian, China). After collecting the positive bacterial clones, plasmids were extracted using a NucleoSpin Plasmid Kit (Macherey‐Nagel), and were sequenced using a custom service provided by LGS Genomics GmbH. The obtained sequences were assembled and analyzed using the CLC Main Workbench (Version 6.9.2; Qiagen) software. At least three ITS1 and ITS2 sequences along with the 5.8 rDNA sequence were obtained for each duckweed clone to produce a consensus sequence. The obtained consensus sequences were deposited in GeneBank (for accession numbers see Table S1).

### Phylogenetic analysis

The maximum‐likelihood phylogenetic trees for phylogenetic analysis of ITS and plastid sequences were constructed applying the NGPhylogeny web service (https://ngphylogeny.fr) (Lemoine et al., 2019) using MAFFT Multiple Sequence Alignment (Katoh & Standley, 2013) and the PhyML algorithm with Smart Model Selection (Guindon et al., 2010). Cleaning aligned sequences was made by utilizing BMGE tools (Criscuolo & Gribaldo, 2010). Bootstrap support (BS) (Felsenstein's bootstrap proportions [FBPs] + transfer bootstrap expectation [TBE]) was estimated with 100 bootstrap replicates. iTOL (https://itol.embl.de) was used for displaying and annotating the generated phylogenetic trees (Letunic & Bork, 2021).

### Tubulin‐based polymorphisms (TBP) profiling

The capillary electrophoresis tubulin‐based polymorphism (CE‐TBP) method was carried out according to the procedure defined by Braglia et al. (2023). The first and second intron amplifications from 30 ng of genomic DNA were performed twice per clone. Control PCR reactions, without DNA template, were always included in any experiment. The signal intensity of the PCR amplicons was checked on a 2% agarose gel, and the diluted samples were prepared for the capillary electrophoresis separation according to Braglia et al. (2016). Data analysis and collection were performed using the Gene Mapper Software v.5.0 tools (Thermo Fisher Scientific, Inc., Waltham, MA, USA) according to the parameters defined by Braglia et al. (2023). The size (in base pairs) and the height (in relative fluorescence units = RFUs) of each peak composing the CE‐TBP pherogram of each clone were collected into Microsoft Office Excel files. The numerical data sorted according to the peak size was considered when comparing the CE‐TBP profiles of the analyzed samples. A presence/absence matrix (1/0 respectively) was obtained by peak scoring of both intron regions (first and second). The Jaccard's index genetic similarities among clones were estimated, and a neighbor‐joining (NJ) dendrogram, with 1000 replicates (bootstrap analysis), was computed using the open‐source software package Past v.4.14 (Hammer et al., 2001).

### Genome size measurement

For flow cytometric genome size measurements fresh leafy fronds were chopped together with an appropriate amount of leaf tissue of one of the reference standards (*Raphanus sativus* cultivar “Voran” Gatersleben Genebank accession number: RA 34 [1.11 pg/2C]; *Lycopersicon esculentum* Mill. convar. *infiniens* Lehm. var. *flammatum* Lehm., cultivar “Stupicke Rane,” Gatersleben Genebank accession number: LYC 418 [1.96 pg/2C] or *Glycine max* (L.) Merr. convar. *max* var. *max*, cultivar “Cina 5202,” Gatersleben Genebank accession number: SOJA 392 [2.21 pg/2C]) with a sharp razor blade and using the “CyStain PI Absolute P” nuclei extraction and staining kit (Sysmex‐Partec, Görlitz, Germany) according to the manufacturer's instruction. After filtering the suspension through *a* 50 μm mesh (CellTrics, Sysmex‐Partec), samples were analyzed on a CyFlow Space flow cytometer (Sysmex‐Partec). The DNA content (pg/2C) was calculated based on the values of the G1 peak means of sample and reference species, and the corresponding genome size (Mbp/1C) according to Dolezel et al. (2003). Genome size measurements were done in at least three replicates for each tested accession.

### Mitotic chromosome preparation

The plants were grown in SH medium (Schenk & Hildebrandt, 1972) with 0.5% sugar until new daughter fronds had developed. The fronds were collected, treated in 0.02 m 8‐hydroxyquinoline at 37°C for 1 h, and then fixed in fresh 3:1 (absolute ethanol:acetic acid) for 24 h. The fixed samples were washed twice in 10 mM Na‐citrate buffer pH 4.6 for 10 min each, before and after softening in 2 ml PC enzyme mixture [2% pectinase and 2% cellulase in Na‐citrate buffer] for 60 min at 37°C, before maceration and squashing in 60% acetic acid. After freezing in liquid nitrogen, the slides were treated with pepsin (50 μg pepsin ml^−1^ in 0.01 N HCl, 5 min at 37°C), post‐fixed in 4% formaldehyde in 2×SSC for 10 min, rinsed twice in 2×SSC, 5 min each, dehydrated in an ethanol series (70, 90 and 96%, 2 min each), and air‐dried.

### Probe labeling

The DNA probes specific for the genomes of diploid *Le. aequinoctialis* and *Le. perpusilla*, respectively, were prepared following the procedure described by Hoang and Schubert (2017). First, genomic DNA isolated from *Le. aequinoctialis* (2018) and *Le. perpusilla* (BOG0007) was sonicated to obtain fragments of 500–1000 bp. This DNA was used as a template for labeling with specific fluorescent dyes. The DNA was subjected to nick‐translation using the Alexa594 NT Labeling Kit and the Atto488 NT Labeling Kit (Jena Bioscience, Jena, Germany). After precipitation with 96% ethanol, probe pellets were dissolved in 100 μl hybridization buffer (50% [v/v] formamide, 20% [w/v] dextran sulfate in 2×SSC, pH 7).

### Genomic *in situ* hybridization (GISH)

GISH with genomic DNA of *Le. aequinoctialis* and *Le. perpusilla* was applied to well‐spread chromosome preparations of the species of probe origin and those of presumed hybrids. Probes were denatured at 95°C for 5 min and chilled on ice for 10 min before adding 10 μl of each probe per slide. Then, the mitotic chromosome preparations were denatured together with the probes on a heating plate at 80°C for 3 min, followed by incubation in a moist chamber at 37°C for at least 16 h. Post‐hybridization washing and signal detection were done as described (Lysak et al., 2006) with minor modifications.

### Super‐resolution microscopy

To analyze the ultrastructure of chromatin beyond the classical lateral Abbe‐Rayleigh limit of ~250 nm, spatial structured illumination microscopy (3D‐SIM) was performed to achieve a lateral resolution of ~120 nm (super‐resolution, attained with a 488 nm laser). We used an Elyra 7 microscope system equipped with a 63×/1.4 Plan‐Apochromat objective and the ZENBlack software (Carl Zeiss GmbH). Image stacks were captured separately for each fluorochrome using 405 nm (DAPI), 488 nm (Atto488) and 561 nm (Alexa594) laser lines for excitation and appropriate emission filters (Kubalová et al., 2021; Weisshart et al., 2016). Zoom‐in sections are presented as single slices to detect the subnuclear chromatin structures at the super‐resolution level. Chromosome counting was done within the spatial image stacks.

## AUTHOR CONTRIBUTIONS

LM, IS, AS, LB, did conceptual work. AS, LB, JF, VS, PTNH, YL, SG, GC performed and evaluated experiments. AS, IS, LM, LB wrote the paper. All authors edited and approved the manuscript.

## CONFLICT OF INTEREST

All authors declare no conflict of interest.

## Supporting information


**Figure S1.** Alignment of the *atpF*‐*atpH* spacers of *Lemna aequinoctialis*, *Le. aoukikusa*, *Le. perpusilla*, and *Le. tenera* clones. Matching residues are shown as dots. Gaps are highlighted in light blue.
**Figure S2.** Alignment of *psbK*–*psbI* spacers of *Lemna aequinoctialis*, *Le. aoukikusa*, *Le. perpusilla*, and *Le. tenera* clones. Matching residues are shown as dots. Gaps are highlighted in light blue.
**Figure S3.** Alignment of ITS1‐5.8S‐ITS2 sequences of *Lemna aequinoctialis*, *Le. aoukikusa*, *Le. perpusilla*, and *Le. tenera* clones. Matching residues are shown as dots. Gaps are highlighted in light blue. Clones with two ITS types highlighted in magenta (NGY128) and orange (9648).

**Table S1.** The list of duckweed accessions and research conducted on them.

**Table S2.** List of primers used in this study.

**Table S3.** Schematic representation of the numerical output of TBP profiles for each analyzed accession.

## Data Availability

The sequences of all cloned PCR fragments are available in GenBank (accession numbers PQ859380–PQ859435, PQ869513–PQ869612).
