# Supplementary material for: Genome diversity and evolution of the duckweed section Alatae comprising diploids, polyploids, and interspecific hybrids
Source: Plant J. 2025 Apr 23;122(2):e70158. doi: 10.1111/tpj.70158 (PMC12018649; doi:10.1111/tpj.70158)
Supplement: Supplementary file 3 — Table S2. List of primers used in this study. [file TPJ-122-0-s003.pdf]

Table S2 List of primers used in this study.

Primer ID	Sequence, 5'→3'	Direction	Target	Used for
DW18S-F1	CCGCCCGCGACGTCGCGA	Forward	18S rDNA	ITS1-5.8S rDNA-ITS2 amplification
DW25S-R1	TTATATGCTTAAACTCGGCG	Reverse	25S rDNA	ITS1-5.8S rDNA-ITS2 amplification
TBP-Fex1	AACTGGGCBAARGGNCAYTAYAC	Forward	β-tub exon1	β-tub gene intron 1 amplification
TBP-Rex1	ACCATRCAYTCRTCDGCRTTYTC	Reverse	β-tub exon2	β-tub gene intron 1 amplification
TBP-Fin	GARAAYGCHGAYGARTGYATG	Forward	β-tub exon2	β-tub gene intron 2 amplification
TBP-Rin	CRAAVCCBACCATGAARAARTG	Reverse	β-tub exon3	β-tub gene intron 2 amplification
